# Technological Advances in Clinical Definition and Surveillance Methodology for Surgical Site Infection Incorporating Surgical Site Imaging and Patient-Generated Health Data

**DOI:** 10.1089/sur.2019.153

**Published:** 2019-09-10

**Authors:** Robert G. Sawyer, Heather L. Evans, Traci L. Hedrick

**Affiliations:** ^1^ Department of Surgery, Western Michigan University Homer Stryker M.D. School of Medicine, Kalamazoo, Michigan.; ^2^ College of Engineering and Applied Sciences, Kalamazoo, Michigan.; ^3^ Department of Surgery, Medical University of South Carolina, Charleston, South Carolina.; ^4^ Department of Surgery, University of Virginia, Charlottesville, Virginia.

**Keywords:** patient-generated health data, surgical site infection

## Abstract

***Background:*** Surgical site infection (SSI) continues to be a common and costly complication after surgery. The current commonly used definitions of SSI were devised more than two decades ago and do not take in to account more modern technology that could be used to make diagnosis more consistent and precise. Patient-generated health data (PGHD), including digital imaging, may be able to fulfill this objective.

***Methods:*** The published literature was examined to determine the current state of development in terms of using digital imaging as an aide to diagnose SSI. This information was used to devise possible methodology that could be used to integrate digital images to more objectively define SSI, as well as using these data for both surveillance activities and clinical management.

***Results:*** Digital imaging is a highly promising means to help define and diagnose SSI, particularly in remote settings. Multiple groups continue to actively study these emerging technologies, however, present methods remain based generally on subjective rather than objective observations. Although current images may be useful on a case-by-case basis, similar to physical examination information, integrating imaging in the definition of SSI to allow more automated diagnosis in the future will require complex image analysis combined with other available quantified data.

***Conclusions:*** Digital imaging technology, once adequately evolved, should become a cornerstone of the criteria for both the clinical and surveillance definitions of SSI.

## Statement of the Problem

Surgical site infection (SSI) continues to be a major cause of morbidity among surgical patients and is associated with the greatest financial burden among all health-care–associated infections [1]. Because of this, regulatory scrutiny related to SSI has become intense in terms of measuring both process measures and outcomes. In some situations, financial penalties might be levied upon poor performers.

Underlying this well-intentioned enthusiasm for decreasing rates of SSI, a fundamental problem exists. The current gold standard definition of SSI, developed by the U.S. Centers for Disease Control and Prevention (CDC), is now 27 years old and does not take advantage of new technology, such as the electronic health record, that could make detecting SSI easier and more consistent [2]. In addition, currently used schema to diagnose SSI are somewhat subjective, including the CDC's: “Diagnosis…made by a surgeon or attending physician [2],” or certain elements of the numerical Additional treatment, Serous discharge, Erythema, Purulent exudate, Separation of the deep tissues, Isolation of bacteria, and the duration of inpatient Stay (ASEPSIS) scoring system, such as assigning points for the use of antibiotics to treat cellulitis, a subjective intervention [3]. Finally, the ideal process of having an unbiased, trained observer examine surgical sites to determine the presence of SSI is impractical and prohibitively expensive. Instead, many health systems rely on self-reporting of SSI by surgeons, an approach that is problematic at best. Because of these vagaries, multiple definitions of SSI are used, both for clinical care and surveillance purposes [4].

## Differentiating Clinical and Surveillance Definitions

Any discussion of definitions of health-care–associated infections and how modern technology can be leveraged to improve them must begin with an acknowledgment that clinical and surveillance SSI definitions, although ideally identical or similar, are used for different purposes in the healthcare system. Clinical definitions are formulated to allow ideal patient care. Because an SSI in an individual patient leads to treatment, all patients are dichotomized as having or not having an SSI, even though there may be uncertainty in the mind of the treating clinician. The clinical diagnosis of an SSI also has the advantage of including an almost endless number of variables in the mind of the healthcare provider: all available laboratory tests, imaging studies, physical examination, and how the incision has changed over time. On the other hand, surveillance definitions must, for efficiency's sake, rely on a limited number of data points.

Because the purpose of surveillance is to detect major changes in disease over time (rather than to treat individual patients), surveillance definitions of SSI are optimized to produce data at the population level. As a result, sampling can be used to estimate rates of SSI in a population. Information is not necessarily collected from every patient, operation, surgeon, or subspecialty, introducing some bias to the results. For example, patients who are lost to follow-up or receive care at a venue not affiliated with the place of surgery will yield missing data. These data subsequently must be extrapolated to any unstudied population, and this may result in inaccuracies. For example, in one hospital an increase in SSI rates caused by resistant pathogens among colorectal surgery patients (a heavily studied population) may or may not imply a similar increase in infections among head and neck surgery patients (a less studied group).

Furthermore, because of limited resources, surveillance SSI definitions should be easily applied in a time-efficient manner and be consistent between observers. Ideally, by reducing subjectivity, they should also decrease the frequency of intentional or unintentional underreporting based on concerns over financial penalties for SSI. The actual requirements for clinical and surveillance definitions of SSI, therefore, are different, and whether or not clinical and surveillance definitions can ever be identical is far from clear. Information technology holds the promise of being able to narrow these differences by introducing new methods of automated and objective analysis.

## Goals for an Ideal SSI Definition Incorporating Imaging

One form of information technology that can rapidly improve clinical care and surveillance of patients with SSIs is digital image capture and analysis (potentially automated). Even more intriguing, considering the common availability of cameras on mobile phones and other devices, is the use of patient-generated data (images) to help in the diagnosis and management of SSI. Including imaging and analysis in the definition SSI should be based on several identifiable goals. First, an SSI should be identifiable in a patient almost regardless of the patient's care site or the personnel immediately available. For example, an SSI should be able to be diagnosed similarly whether in the surgeon's office or an urgent care center. Second, the technology should allow for a more rapid and accurate clinical diagnosis, in order to speed care, whether the incision is open or antibiotics started. Similarly, imaging should decrease the frequency with which patients present to medical care with surgical site concerns, as long as SSI can be ruled out reliably. Third, from a surveillance perspective, incision imaging should be able to be incorporated into surveillance workflow more quickly than waiting for information derived from physician offices or clinics. This acceleration of the process should lead to a more rapid identification of changes in infection rates overall or outbreaks with specific, targeted pathogens. Finally, high-quality image analysis incorporating artificial intelligence should reduce or eliminate the subjectivity in the diagnosis of SSI based on inspection.

## Prior Literature on the Use of Imaging in the Diagnosis of SSI

With several of the above goals in mind, some investigators have begun to investigate the use of imaging to diagnose SSI. Gunter et al. [5] reported on a series of 40 vascular surgery patients who used their mobile devices to transmit images of their incisions to investigators. Combined with questionnaire data, seven surgical site complications were diagnosed, and patient participation was good.

Although image capture appears practical, without automated image analysis, one substantial concern is the potential for variability in diagnosis based on the clinician viewing the images, i.e., interrater agreement. Van Ramhorst et al. [6] tested four surgeons, having them analyze incision photographs to make the diagnosis of SSI. Specificity of diagnosis was 97%, and κ for interrater agreement ranged from 0.43 to 0.76, showing modest to good reliability. Lepelletier et al. [7] used a vignette-based method including photographs to assess interrater agreement among 20 different specialties. Although interrater agreement among similar specialists was reasonable (maximum κ of 0.73 for infectious diseases practitioners), it was poor between specialties, at 0.47 overall. Hedrick et al. [8] obtained serial imaging of incisions after colorectal surgery in 171 subjects. Three attending surgeons analyzed the images and other data, calculated an ASEPSIS score [3], and judged the presence or absence of SSI. Although the overall rates of SSI were somewhat similar (ranging from 6%–14% between the evaluators), the overall κ statistic was modest at 0.55. Using smartphone digital images, Wiseman et al. [9] analyzed specific incision characteristics for interrater reliability and found that agreement was highest for necrosis and dehiscence, but lowest for redness and ecchymosis. The authors found that although the interrater variability varied between specific incision characteristics, the reliability for determining treatment recommendations was high. Finally, Sanger et al. [10] used Web-based simulation surveys to query 83 surgeons asking them to diagnosis SSI based on history and descriptions of the incision, then provided them with incision photographs. Although the inclusion of photographs increased the diagnostic specificity from 77% to 92%, the participant mean diagnostic accuracy was only 75%, with a wide interquartile range of 66%–92%. Importantly, however, the addition of photographs dramatically decreased overtreatment including recommendation for emergency department visit and prescription of antibiotics.

## Imaging as One Component of a Definition

As noted in the previously cited article by Sanger et al. [10], incision imaging enhances the use of other data points to diagnose SSI but does not replace them. As such, similar to other SSI definitions in the past, multiple parameters will be needed to construct an ideal definition. Potential origins of these parameters include demographics, medical history (immunosuppression), surgical history, symptoms, vital signs, laboratory values, and potentially radiologic imaging studies. A major consideration when including or excluding these parameters is the ease with which they can be extracted from a robust electronic health record. An accurate definition that relies on easily portable data, such as laboratory values and demographics, would be preferable to one requiring either human, manual, data extraction, or complex analytical techniques, such as natural language processing of radiology reports.

Several groups have already analyzed the ability of electronic health record-derived data to diagnose SSI. Hu et al. [11] developed several machine-learned models using data from the electronic health record to predict the likelihood of SSI. Over a period of 33 months, 6,258 procedures were performed and 405 SSIs were identified based on National Surgical Quality Improvement Program (NSQIP) data. The models built had excellent negative predictive values generally above 95%. Focusing on patient-generated data, Macefield et al. [12] used a validated three-phase, iterative approach to develop a 16-item questionnaire for patients as part of post-discharge SSI surveillance. The questions are presented in the patient's voice and generally assess the incision, need for antibiotics or incision management, and interactions with the healthcare system. This group has further tested prospectively the questionnaire in nearly 600 patients, noting an outstanding discrimination for SSI with a receiver operating characteristic area under the curve C statistic of 0.91 [13]. A key limitation is whether widespread incorporation of a 16-item questionnaire will be practical outside of a research setting.

## Using Mobile Health and Patient-Generated Health Data for Image Analysis

Patient-generated health data (PGHD) captured via mobile health (mHealth) for SSI surveillance presents a novel opportunity to leverage the data produced for research. Mobile health tools for SSI surveillance allow for the collection of post-operative incision photographs taken by patients after hospital discharge [14]. The process of turning a digital image (potentially coming from any number of sources) into an objective piece of data is one key development that is necessary to evolve the definition of SSI into one that both includes imaging and can be captured from the electronic health record. Obviously, this will entail complex computerized image analysis, resulting in either an overall assessment of a incision (infected or not), or multiple measures of discrete incision characteristics (erythema, discharge, separation, etc.), or both. The process to build such models is arduous and will require thousands of images for training, although such libraries are already in process [15]. However, once built, such a mechanism could be further trained on an ongoing basis to refine this variable further.

**FIG. 1.**
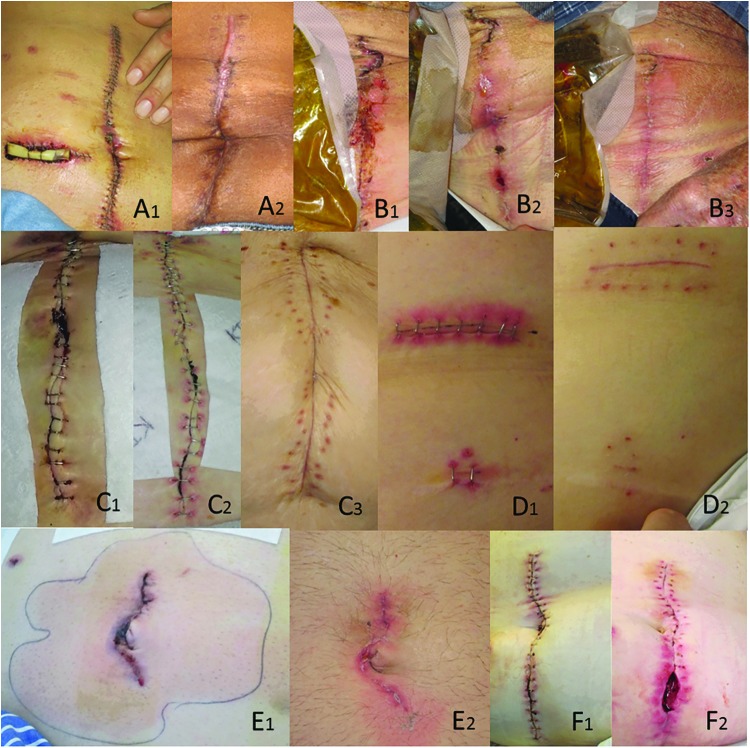
Examples of incision images demonstrating diverse photographic data on patients who exhibit normal incision healing, as well as those who experience complications of incision healing. Patients are labeled A–F with numbers representing sequential images of the same incision over time. Color image is available online.

**FIG. 2.**
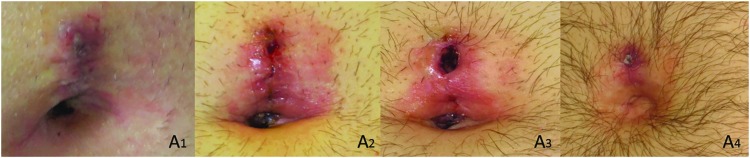
Patient-generated serial post-operative incision images of the same patient at four subsequent time points (A1–A4) demonstrating the ability to visualize and analyze incisions over time. Color image is available online.

Ideally a database incorporating a diverse group of images from a collaborating network of institutions would be formed for storage and analysis. Diverse photographs/samples are needed to address inherent analysis biases introduced by homogeneity of patient populations contributing to the database. It is important to capture photographic data on patients who exhibit normal incision healing, as well as those who experience complications of incision healing, inclusive of those who develop SSI (Fig. 1). Production of serial post-operative incision images via PGHD presents the opportunity for a large volume of data generation, and the ability to visualize and analyze incisions over time (Fig. 2). Current practice in SSI monitoring, based in clinic/hospital settings, allows for limited data generation, and rarely the production of serial images. Serial images could impact current understandings of the progress of normal versus abnormal post-operative incision healing and provide more robust and diverse training sets for clinicians and researchers.

Additional value of an incision image database lies in the potential to utilize machine learning and computer vision methods to create algorithms for SSI surveillance, for example, to determine which patients are progressing toward abnormal incision healing and may be at increased risk of SSI. Analysis of serial incision photographs could also add to the knowledge base of what additional criteria may be needed to assess for SSI using mHealth tools.

Fortunately, image analysis already has precedent in several related fields. For example, Landa et al. [16] developed software to analyze traumatic wound images and characterize the surface area, granulation, slough, and necrosis in each wound, testing the program on 20 different wound photographs. Interrater reliability was highest for surface area (κ = 0.99), followed by granulation (0.76), slough (0.67), and necrosis (0.22). A similar system could be devised to analyze characteristics of post-surgical incisions, such as skin edge apposition, erythema, and discharge. However, a barrier to mHealth for SSI surveillance is that patients take and submit photographs using devices of varying quality. Device angle, lighting, and distance from the wound can all impact the quality and usability of photos taken. Adequate patient training will be necessary to ensure high-quality photographs.

## How SSI Definition Information May Be Incorporated into Clinical Care and Surveillance

With an updated definition of SSI incorporating enhanced data capture from the electronic health record, PGHD integration of image capture, and computer image analysis, it is reasonable to contemplate how a more automated surgical site assessment could be used. First, especially if an algorithm developed through machine learning is utilized, the output from such an assessment would yield the probability of presence of SSI. From a clinical standpoint, scores at the extremes would be easy to manage and dichotomized into “no therapy” versus “intervention” (incision opening and/or antibiotics). Scores in the middle, for example in the 20%–80% probability of infection range, would doubtlessly generate management algorithms, probably incorporating some form of re-evaluation, re-imaging, laboratory studies, or radiologic imaging.

Similarly, scalar rather than dichotomous results would affect how surveillance data are interpreted and used. On the one hand, all surveillance cases could be dichotomized as infection yes or no (as is the current practice), based on an agreed-upon risk such as 90%. Alternatively, a mean SSI risk score could be calculated for each procedure of interest, and institutional outcomes compared against this standard. This concept is similar to that underlying the ASEPSIS score [3], which measures both presence and severity of infection. For example, for open colectomy, the mean risk of SSI based on a machine learned algorithm might be 16%, a figure different than the percentage of patients treated for SSI. The potential advantage of this approach would be incisions with more severe stigmata of infection (higher calculated probability of infection) would be weighted more heavily than infected incisions with relatively minor findings (lower probability of infection). Currently for surveillance, other than differentiating between superficial incisional, deep incisional, and organ/space infections, all SSI no matter the local severity, are counted the same.

## Conclusion

Although current surveillance standards inform clinical practice, gaps exist between accepted national guidelines [2,3] and how they are applied in the context of care delivery [17,18]. These challenges persist for a variety of reasons including lack of consensus around what criteria are both sufficient and necessary for diagnosing SSI in the clinical context, and inconsistent interpretation of those criteria that may vary by the individual applying them [7,8,19-21]. Definitions of SSI are neither agreed upon uniformly nor reported consistently across research projects [12,16]. Furthermore, currently available mHealth tools for monitoring SSI have the potential to transform the definition and surveillance methodology of SSI. Patient incision photographic data and mHealth should be leveraged to address these issues, increase the breadth and depth of what is known about surgical incision healing, and inform the development of best practices and standards for the surveillance and clinical diagnosis of SSI.
